# Immunogenicity and toxicity of AAV gene therapy

**DOI:** 10.3389/fimmu.2022.975803

**Published:** 2022-08-12

**Authors:** Hildegund C. J. Ertl

**Affiliations:** Ertl Laboratory, Vaccine Center, The Wistar Institute, Philadelphia, PA, United States

**Keywords:** AAV, T cell responses, B cell responses, innate immune responses, serious adverse events

## Abstract

Gene transfer using adeno-associated viral (AAV) vectors has made tremendous progress in the last decade and has achieved cures of debilitating diseases such as hemophilia A and B. Nevertheless, progress is still being hampered by immune responses against the AAV capsid antigens or the transgene products. Immunosuppression designed to blunt T cell responses has shown success in some patients but failed in others especially if they received very high AAV vectors doses. Although it was initially thought that AAV vectors induce only marginal innate responses below the threshold of systemic symptoms recent trials have shown that complement activation can results in serious adverse events. Dorsal root ganglia toxicity has also been identified as a complication of high vector doses as has severe hepatotoxicity. Most of the critical complications occur in patients who are treated with very high vector doses indicating that the use of more efficient AAV vectors to allow for dose sparing or giving smaller doses repeatedly, the latter in conjunction with antibody or B cell depleting measures, should be explored.

## Introduction

Gene transfer using vectors based on adeno-associated viruses (AAV) has achieved lessening of symptoms or even cures of diseases caused by single gene defects such as Leber’s congenital amaurosis, a congenital form of blindness (1), or hemophilia B due to loss-of-function mutations within the gene encoding coagulation factor 9 (2). Although AAV vectors are poorly immunogenic and persist in mice, dogs, and nonhuman primates for many years (3–5), humans injected with large doses mount T and B cell responses against the AAV capsid or even the transgene product. T cells can lead to rejection of transduced cells (6, 7) while B cells if they produce AAV neutralizing antibodies prevent successful reapplication of the vector (8). Even worse if antibodies neutralize a soluble transgene product, they may interfere with traditional protein therapies (9).

Immune mediated rejection of AAV vector transduced cells can in part be prevented by immunosuppressants given during or shortly after gene transfer (2). Immunosuppressive drugs have improved the outcome of AAV-mediated gene transfer, but some patients fail to respond and still reject the AAV-transduced cells (10–12) while others do not need the drugs but receive them as it still impossible to predict which patients will or will not mount destructive immune responses.

Although we have gained over the last two decades a better understanding of innate and adaptive immune responses to natural infections with AAV or AAV vector-mediated gene transfer using different serotypes applied at various doses to different organs some basic questions remain unanswered.

**Figure 1:**
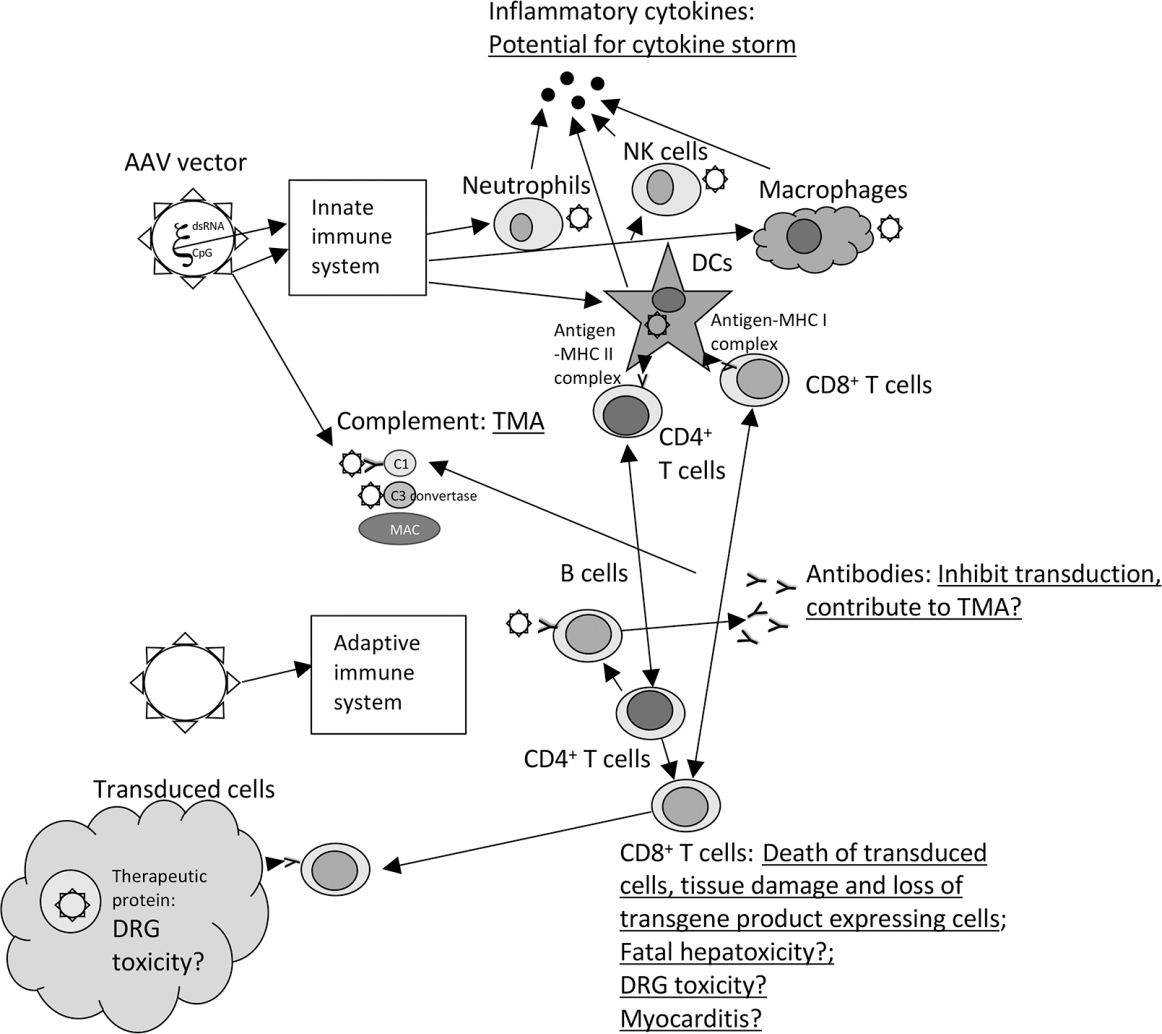
Immune responses and toxicity to AAV vectors. The graph shows the different type of immune responses that are elicited upon injection of AAV vectors. Toxicities are underlined and listed next to the components of the immune responses that contribute or may (?)contribute to the adverse events. The following abbreviations are used within the figure: CpG, unmethylated CpG motifs; C1 and C3, complement factors 1 or 3; DCs, dendritic cells; DRG, Dorsal root ganglia, dsRNA, double stranded RNA; MAC, membrane attack complex; MHC, major histocompatibility complex; NK cells, natural killer cells, TMA, thrombotic microangiopathy,? – remains to be tested in more detail.

This manuscript reviews the different types of immune responses that are elicited by AAVs and how they translate into lack of efficacy or even worse toxicity (**Figure 1**).

## Basic biology of AAVs and AAV vectors

AAVs are dependoviruses that naturally infect primates and other species where they can replicate with the assistance of a helper virus such as an adenovirus. As a genus they have a wide host cell range for both resting and dividing cells although different serotypes of AAVs or AAV vectors have a more restricted tropism; for example, in humans AAV1 vectors have high tropism for muscle while AAV2 vectors readily infects lung and brain cells (13). Tropism may differ between species: AAV8 vectors more efficiently infect mouse than human hepatocytes (14). AAV infections are asymptomatic, and the virus persist for the lifetime of an individual. Nevertheless, AAVs are immunogenic as they stimulate detectable B and T cell responses (15). As induction of long-lived adaptive immunity depends on antigen-presenting cells that require prior maturation driven by inflammatory signals AAV infections must be able to trigger innate immune responses that upon a natural infection may in part be driven by the helper virus.

AAVs carry a single stranded DNA genome with two open reading frames (ORFs) encoding regulatory proteins as well as the capsid antigens. The genome is flanked by invert terminal repeat (ITR) sequences. In AAV vectors the two ORFs are deleted and replaced with an expression cassette for a therapeutic protein or RNA; during viral production the deleted sequences as well as essential sequences from a helper virus are provided in trans. Upon injection antigens from the capsids of AAV vectors are present in the inoculum but they, unlike the transgene product, are not actively produced by the transduced cells. This is turn limits the duration of presentation of capsid antigens to the immune system till the proteins are degraded, which according to clinical trial results may take months (16).

## Immunology 101 as it pertains to AAVs and AAV vectors

### Innate responses

Viruses or viral vectors carry either within their antigens or their genome pathogen-associated molecular patters (PAMP), which are absent in mammals and therefore recognized as threats by so-called pathogen recognition receptors (PRR), which are located on the cell surface, within endosomes or the cytosol (17). Expression of certain types of PRRs such as Toll-like receptors (TLR) is commonly cell-type specific; for example, TLR2 is expressed by macrophages but not dendritic cells while TLR3 has the opposite expression pattern (18). Binding of a PAMP to a PRR initiates a signaling cascade that ultimately results in activation of transcription factors such as nuclear factor kappa B (NF-kB), activator protein 1 (AP-1), interferon regulatory factors (IRF)-3 and -7 (19). This initiates production of inflammatory mediators such as cytokines and chemokines, maturation of dendritic cells into professional antigen presenting cells and activation of other cells of the innate immune system such as natural killer cells, macrophages and neutrophiles. Viruses can also elicit inflammatory responses by initiating an unfolded protein response that is typically triggered as a reaction to endoplasmic reticulum (ER) stress. AAV vectors expressing for example the coagulation factor VIII trigger an ER stress response due to overexpression of this protein; ER stress in turn is likely to promote activation of AAV- or FVIII-specific immune responses (20). In most cases innate immune responses are non-antigen-specific, their activation is short-lived, and they lack ‘memory’ so that a 2^nd^ exposure to the same pathogen elicits very similar responses.

Complement, which is part of the innate immune system but relies in some respects on effector molecules of the adaptive immune system provides a crucial defense mechanism against viruses. It can kill or neutralize viruses directly, it can aggregate viruses, promote their phagocytosis by leukocytes, and in concert with virus-specific antibodies kill infected cells thereby stopping production of new viral progeny (21). Three pathways can activate complement to form through several step the so-called membrane attack complex (MAC) (22). In the classical pathway antigen-antibody complexes activate the C1 complex, which then splits both C2 and C4 into a and b. C4b and C2b form a complex called C3 convertase that cleaves C3 into a and b; the latter together with the C4bC2b complex digests C5 into a and b. C5b binds to C6, C7, C8, and C9 to form the MAC. In the lectin pathway viral carbohydrates bind to the mannose binding lectin (MBL)/ficolin- mannose-associated serine protease 1 (MASP) complex, which activates the C1 complex. Some viruses activate the alternative complement pathway. This pathway constantly converts at low levels C3 to C3a and C3b. Normally complement inhibitory factors H and I inactivate C3b preventing full activation of the pathway. Some viruses bind to C3b leading to cleavage of complement factor B into a and b. Bb binds to C3 forming an alternative C3 convertase which can that cleave C3 into a and b which results in full complement activation (23). Activated complement initiates an inflammatory reaction (24), serves as a powerful attractant for neutrophiles, triggers coagulation pathways (25), damages endothelial cells (26) and activates platelets, which can lead to microvascular injuries and thrombus formation (27). AAV vectors bind to complement factor C3 but as was shown for AAV8 vectors also elevate complement inhibitory factors H and I; it is therefore assumed that AAV vectors do not directly activate the alternative complement pathway (28).

### Adaptive immune responses

Antigen-specific adaptive immune responses come up more slowly than innate responses and depending on the antigenic load, site of antigen exposure and strength of the inflammatory reaction can take one to several weeks before they become detectable in blood. B cells recognize linear or conformational epitopes on soluble or surface-bound antigens. Naïve B cells initially differentiate into short-lived plasma cells, which mainly produce IgM antibodies that have not undergone affinity maturation. With the help of follicular T helper cells antigen-exposed B cells then form germinal centers where they undergo class-switching and affinity maturation followed by differentiation into long-lived plasma cells and memory B cells. Long-lived plasma cells home to lymphatic tissues and bone marrow, the latter provides them with a niche where they can survive for decades. Memory B cells mainly stay within lymph nodes or spleens. Upon reencounter of their antigen, they either proliferate and undergo additional rounds of affinity maturation or they assume effector function and start secreting antibodies. Memory antibody responses typically come up faster than primary responses, peak responses are higher and more sustained, and antibodies show increased avidity to their cognate antigen. They are also less dependent on T cell help, which is essentially for induction of primary affinity matured antibody responses (29–31).

T cell are activated by small peptides that a generated upon intracellular degradation of viral proteins. Peptides bind to MHC molecules and are then transported to the cell surface where those associated to MHC class I molecules are recognized by CD8^+^ T cells while peptides bound to MHC class II molecules trigger a CD4^+^ T cell response. MHC class I molecules are present on most cells while MHC class II molecules are only expressed by cells of the immune system such as macrophages, dendritic cells, and B cells. Upon activation depending on the type of the inflammatory reaction and the strength of T cell receptor (TcR) signaling CD4^+^ T cells differentiate into different subsets, i.e., Th1 cells, which promote CD8^+^ T cell responses, Th2 cells,which drive B cell activation, Th17 cells, which induce strong inflammatory responses and activate neutrophiles, Th22 cells, which play a role in protecting skin against infections and regulatory T cells (Tregs), which dampen effector T cell responses and are crucial to maintain tolerance against self-antigens (32, 33).

Activation of naïve CD8^+^ T cells in general requires help from CD4^+^ T cells and mature dendritic cells that present the MHC class I – peptide complexes together with co-stimulators such as CD80 or CD86, which bind to CD28 on T cells. Naïve CD8^+^ T cells can be stimulated by directly infected dendritic cells or for viruses that are unable to infect these crucial antigen presenting cells by a process called cross-presentation (34). CD8^+^ T cells are exquisitely sensitive to antigen and as few as 2-4 MHC class I – peptide complexes can trigger a response (35).

After activation CD8^+^ T cells switch their metabolism towards glycolytic energy production. This switch is essential to generate fully functioning effector T cells (36) as it supports the T cells’ rapid proliferation and provides building blocks for dividing cells and effector molecules. Upon activation CD8^+^ T cells migrate to the infected tissue where they release anti-viral cytokines such as interferon (IFN)-g or lyse antigen-expressing cells through the release of perforin and granzyme B. Once the antigen-expressing cells have been removed, most activated CD8^+^ T cells undergo apoptosis, the remaining cells differentiate into different subsets of memory cells: effector memory cells circulate, they are still partially activated and can commence effector functions immediately without further proliferation; central memory cells home to lymph nodes, they are ‘resting cells’ and upon encounter of their antigen proliferate before they become effector cells. Tissue resident memory cells can be found in nearly all tissues; they provide rapid local protection by immediately assuming effector functions once they encounter their cognate antigen (37).

Chronic infections during which viral loads remain high lead to so-called T cell exhaustion: T cells upregulated co-inhibitory molecules on their surface, change their metabolism from glycolytic energy production to fatty acid oxidation, undergo irreversible epigenetic changes, lose functions, and eventually die (38, 39). Persistent infection where viruses either go into latency or remain present at only very low levels do not lead to T cell exhaustion but can cause an inflated immune response (40).

Recall of memory CD8^+^ T cells is less stringent than activation of a primary response. Memory CD8^+^ T cells can be stimulated by antigen presented without co-stimulators and no longer require T cell help (41). Nevertheless, the memory CD8^+^ T cells’ antigen threshold for activation is slightly higher than that for naïve T cells (42).

## Immune responses and toxicity to AAV vectors

Immune responses and associated adverse events are directly linked to AAV vector doses and in part to mode of application. Low to moderate doses, such as those injected for correction of ocular diseases or hemophilia B using for the latter an optimized transgene product, commonly fail to induce detectable T cell responses although they may stimulate AAV capsid-specific antibody responses (2, 43). AAV vectors given to immune-privileged sites such as the brain or immunosuppressive microenvironments such as the liver (44) are less likely to trigger strong responses than vectors given systemically or to the muscle. It is likely but remains to be proven that age may also play a role as the elderly experience declines in functions of their innate and adaptive immune system (45). Innate responses such as complement activation has resulted in significant toxicity following AAV gene transfer, but most adverse events are linked to adaptive responses. T and B cell responses to transgene products have been reported but more commonly the adaptive immune system reacts to antigens of the AAV capsid. Although most humans, except for very young children, are likely to have immunological memory to AAV capsid antigens it is unknown if recall of these cells is at the root of the problems encountered upon AAV gene transfer or if primary responses to the high vector doses are to blame. This knowledge needs to be gained as it affects which immunosuppressive regimens can blunt the AAV-specific immune responses and allow for sustained transgene product expression. Many of the drugs that are being explored such as rapamycin, an mTOR inhibitor, will block stimulation of naïve cells of the adaptive immune system without affecting recall responses (46, 47). In the same token reducing inflammatory signals by modifying the PAMPs of the AAV vectors may reduce primary responses but will have limited effects on reactivation of memory cells (48).

### Innate responses

Compared to other viruses, AAVs or vectors based on AAVs, only elicit modest inflammatory responses, which can be triggered by TLR9 binding to unmethylated CpG sequences within the vectors’ genome (49), by a PAMP within the capsid proteins that binds to TLR2 (50) and potentially by double stranded RNA, which could interact with melanoma differentiation-associated protein 5 (MDA5, ref. 51), a helicase of the RIG-I-like receptor family, that serves as a cytoplasmic PRR. TLR2 and TLR9 are expressed by monocytes and macrophages. Myeloid dendritic cells are positive for TLR2 while plasmacytoid dendritic cells, a rich source for type I IFN, are positive for TLR9. MDA5 is ubiquitously expressed. Blockade of innate PRR activation by AAV’s PAMPs blunts primary but not secondary immune responses (48, 51–53). The most practiced approach has been removal of CpG rich sequences from the AAV vectors’ genome. Animal studies have shown that a reduction of the vectors’ CpG motifs blunts CD8^+^ T cell responses without modifying B cell responses (52). The ITRs are very rich in CpG sequences that cannot be modified so that complete CpG depletion of the AAV vectors’ genome may not be feasible (54).

The modest innate immune responses that are triggered by AAV vectors might suffice to drive adaptive immune responses but thus far they have not been linked to the direct and early toxicity in clinical trials that are triggered by other viral vectors such as adenovirus vectors. Such vectors, even if used as vaccines at modest doses, elicit within 8-24 hours grade 1-3 systemic reaction in many patients (55) while the high doses used for gene therapy have resulted in death due to hyperactivation of the innate immune system (56). Nevertheless, with higher and higher doses of AAV vectors entering clinical trials, innate immune response may join the list of immune responses that can cause serious adverse events in gene transfer recipients.

### Complement responses

In some young patients, high doses of AAV equal or above 5 x 10^13^ vg/kg have resulted in thrombotic microangiopathies (TMA) with hemolytic anemia, low platelet counts, and hemolytic uremic syndrome (HUS) leading to kidney damage (57–59) even if vectors were given with steroids. The use of AAV vectors for correction of spinal muscular atrophy (SMA) in over 1400 individuals caused 9 cases of TMA in girls between the ages of 4 months to 4 years. In a trial for treatments of Duchenne muscular dystrophy (DMD) injection of an AAV9 vector expressing mini-dystrophin resulted in TMA in 4 out of 15 male recipients between the ages of 7-12 years. In all cases symptoms started 6-12 days after gene transfer. Most patients were treated successfully with plasmapheresis, steroids, hemodialysis, platelet transfusion and eculizumab, a complement inhibitor. Notwithstanding, one patient each in the SMA and the DMD treatment groups died due to TMA complications. Primary TMA is caused by genetic or acquired defects that affect complement regulation. Secondary TMA is caused by activation of complement due to other pathological conditions such as malignancies, drugs, infections, autoimmunity, etc. Infections that can induce secondary TMA include those with influenza viruses, SARS-CoV-2 or B19, a parvovirus that is related to AAV (60, 61).

TMA following AAV gene transfer was associated with pathological activation of complement. Some viruses such as SARS-CoV-2 carry motifs that can activate the lectin pathway of complement (62). This pathway is unlikely to be involved in TMA following AAV gene transfer. The classical pathway of complement activation requires binding of the AAV vector to an antibody. Induction of an IgM response upon AAV gene transfer not only by traditional unswitched B cells but also by B1 and marginal zone (MZ) B cells can occur very rapidly within 3 to 4 days and as IgM is a potent activator of complement these early antibodies could bind AAV and initiate TMA. This would require that by the time antibodies have been produced AAV particles are still accessible in blood or interstitial fluids. Studies in animals with bioluminescent AAV particles have shown that AAV is taken up very rapidly by cells (63) and once the virus has penetrated a cell membrane it is shielded from antibodies casting doubt on this pathway of complement activation in TMA patients. Alternatively, the classical pathway of complement activation could be triggered by pre-existing AAV-binding antibodies; although most AAV gene transfer trials only enroll recipients without detectable AAV neutralizing antibodies, this does not necessarily preclude presence of low levels of binding antibodies or even of naturally occurring IgM that could bind to AAV vectors with low affinity. Activation of the alternative pathway of complement should also be reconsidered. As mentioned above AAV is known to bind to C3 (28) and the simultaneous upregulation of complement inhibitory factor I and H may not suffice to block activation of the pathway by high vector doses. Nevertheless, the kinetics of onset of TMA, which was detected at the earliest by day 6 after gene transfer, argues against activation of the alternative complement pathway by the AAV vector inoculum.

### B cell responses

AAV-specific B cell responses can be induced by natural infections, or they can be stimulated or recalled by AAV gene transfer. But for the potential contribution of AAV-induced antibodies to complement activation and TMA, this immune response does not harm the patient but precludes successful AAV-mediated gene transfer by neutralizing the vectors before they can deliver their payload. Mutations of the vector’s capsid to avoid its neutralization have been tried (64, 65) with albeit limited success as many of the antibodies bind to domains that are crucial for transduction (65). Empty AAV particles have been use as antibody decoys (66), but this approach may increase induction of T cell responses (48). Plasmapheresis including approaches that selectively remove AAV-specific antibodies have allowed for successful AAV transduction in animals with pre-existing neutralizing antibodies (67). Similar results were obtained upon treatment with Imlifidase (IdeS), a streptococcal cysteine protease, that can cleave IgG into F(ab’)_2_ fragments and Fc (68, 69).

Induction of neutralizing antibodies to a secreted transgene product could be very harmful as such antibodies could complicate traditional protein therapy. Stimulation of this response seems to depend on route of vector application. Hepatic transfer of AAV vectors expressing factors VIII or IX, which is the route of administrated for correction of hemophilia, has thus far not resulted in transgene product-neutralizing antibodies, which may in part reflect the inhibitory effects of Tregs, long-lasting protein therapy that resulted over time in tolerance and the careful selection of patients excluding those with a history of inhibitor formation (70). In contrast at least in animals, intramuscular injection of an AAV vector expressing F.IX resulted in formation of inhibitors (71).

Animal studies showed that primary antibody responses to a transgene product can be blocked or at least blunted by immunosuppressive treatments with for example rapamycin and ibrutinib (72). Nevertheless, immunosuppression would be required for lengthy periods of time or even for the lifespan of the gene transfer recipients, which considering the drugs’ impact on sensitivity to infections may not be a viable option.

### T cell responses

In early gene therapy trials for correction of hemophilia B, AAV vector recipients initially developed levels of F.IX that sufficed to lessen their symptoms but then about 4 weeks later developed a subclinical transaminitis that was accompanied by increases in circulating AAV-specific T cells and loss of transgene product expression (6). T cells were further characterized as belonging to the CD8^+^ T cell subset and it was thought that their reflected a recalled memory response of T cells that had been induced by a previous natural infection (7). Subsequent trials carefully monitored the patients after gene transfer and treated increases in liver enzymes with steroids which abrogated T cell responses and allowed for sustained F.IX expression (2, 73). In trials for neuromuscular diseases, which require substantially higher doses of AAV vectors given intravenously, hepatotoxicity was more severe in some patients. About a third of the recipients of Onasemnogene abeparvovec, an FDA approved AAV9 vector therapy for SMA, showed liver damage associated with an inflammatory reaction comprised mainly of CD8^+^ T cells (74). Thus far all patients recovered after treatment with steroids (74, 75).

Although CD8^+^ T cells are the most likely cause for the liver damage upon systemic AAV gene transfer, the hypothesis that the effector cells are derived from a memory cell pool should be revisited. In most hemophilia patients, responses come up very slowly, which is more typical for a primary than a secondary T cell response. Furthermore, in most AAV patients, activation of T cells can be blocked by prednisolone given either at the time of gene transfer or once transaminases increase. Studies with other antigens have shown that steroids act by affecting the T cells’ ability to switch to glycolytic energy production; this blocks activation of naïve CD8^+^ T cells and memory CD8^+^ T cells with low but not high affinity receptors (76). Considering that the CD8^+^ T cell response in AAV gene transfer recipients is driven by limited amounts of antigen derived from the capsid proteins of the input virus particles that degrade slowly over several weeks to months it is hard to imagine that liver toxicity is caused by T cells directed to epitopes, which only bind with low affinity to MHC class I molecules.

### Potential role of immune responses in other adverse events caused by AAV gene transfer

#### Fatal hepatoxicity

In a trial for treatment of X-linked myotubular myopathy, four patients with pre-existing liver disease died several weeks following AAV gene transfer. The patients did not respond to immunosuppressive drugs and their livers showed no evidence of cellular infiltrates, which led to the conclusion that direct vector or transgene product toxicity rather than T cells caused the fatal liver damage. I would like to point out that even a discreet T cell response within the portal and periportal areas of the liver could damage liver circulation (77) that could become fatal in patients with pre-existing liver disease. Any AAV gene transfer trial that requires very high doses of vector should include a careful monitoring of AAV capsid-specific T cells in blood to assist in the analysis of potential serious adverse events and such data are not available for the XLMTM trial.

#### Dorsal root ganglia (DGR) toxicity

Studies in nonhuman primates showed that intrathecal, intracerebroventricular, intra-cisterna magna and to a less extent intravenous transfer of high doses of AAV vectors could result in DGR toxicity, which was not blunted by immunosuppression. Most animals remained asymptomatic but showed upon euthanasia histologic lesions within the central and peripheral nervous system with axonal degeneration, neuronal cell damage accompanied by B and T cell infiltrates (78). Subsequent studies showed that DGR toxicity in nonhuman primates could be blocked by transfer of micro (mi)RNA138 which reduces transgene expression in neurons suggesting that direct toxicity of the transgene product rather than immune mechanisms mediated this adverse event (79).

DRG toxicity was also observed in clinical trials. In one trial 2 patients with familial amyotrophic lateral sclerosis (ALS) and mutations in the gene encoding superoxide dismutase 1 (SOD1) were treated with a single intrathecal infusion of an AAV vector encoding a microRNA targeting SOD1. One patient, who was treated with prednisolone as of the day of gene transfer, showed increases in circulating AAV capsid-specific T cells by about 4 weeks after treatment and concomitantly developed neurological symptoms which according to MRI scans were consistent with DRG toxicity. A second patient was given a more aggressive immunosuppressive regimen; his T cell responses were low and came up later, he developed no neurological symptoms, and his MRI was unremarkable (80–82).

In another trial two patients under immunosuppression were given an rhAAV10 vector expressing survival motor neuron 1 intravenously for treatment of SMA type 2 and again one of the patients developed DRG toxicity (80). It is feasible but at this point unknown if in humans adaptive immune responses such as AAV-specific CD8^+^ T cells contribute to DRG.

### Myocarditis in DMD patients

As described above, an inflammatory milieu is essential to trigger adaptive immune responses and upon AAV gene transfer this is triggered by PAMPs present on or in the AAV vectors. Some disease such as DMD are characterized by increased secretion of pro-inflammatory cytokines by muscle infiltrating leukocyte responding to muscle fiber degeneration (83) which may worsen immune mediated adverse events. This is especially worrisome if the exacerbated inflammation affects the heart muscle. Indeed in a phase Ib trial by Pfizer myocarditis was observed in 2 DMD patients after they received an AAV9 vector expressing mini dystrophin; one of the patients died (https://pharmaphorum.com/news/myocarditis-case-mars-sarepta-dmd-gene-therapy-readout/). In a second trial by Sarepta Therapeutic using an AAVrh74 vector for mini dystrophin 1 out of 20 patients also developed myocarditis (https://investorrelations.sarepta.com/news-releases/news-release-details/sarepta-therapeutics-investigational-gene-therapy-srp-9001), which resolved upon steroid treatment. Although the etiology of AAV vector-induced myocarditis is not yet fully understood is seems feasible that the inflammatory milieu of the damaged muscle tissues in DMD patients favors induction of an immune response to the AAV vectors’ transgene product or alternatively that the added inflammatory reaction induced by the AAV vectors promotes auto-reactive T cell responses to muscle cells.

## Conclusions

Immune system-mediated toxicity continues to challenge successful gene transfer by AAV vectors especially when high doses are required to correct the targeted genetic disease. Immunosuppression, which could be further optimized, has been used successfully to blunt some of the AAV vector-induced immune responses but in other cases has failed. Even more worrisome are some of the more recently described serious or even fatal adverse events such as TMA, fulminant hepatoxicity or myocarditis. TMA is caused by excessive complement activation, but it remains unknown how this pathway is activated upon AAV gene transfer and if genetic variants that affect the complement system played a role. T cell-mediated severe hepatoxicity upon systemic transfer of very high doses of AAV vectors is to be expected by the fatalities observed in patients with pre-existing liver damage may have had a different etiology as remains to be investigated in more detail.

There is no easy pathway to avoid immune-mediated toxicity in response to transfer of very high doses of AAV vectors; our immune system has evolved over the eons to view viruses as threats and responds accordingly by getting rid of them and the cells they have infected. Every war causes collateral damage and the battles the immune system wages against viruses or AAV vectors are no exception. At this stage our focus may have to shift away from the use of excessively high AAV vector doses. Our efforts should concentrate on developing vectors that can more efficiently transduce cells, achieve higher transgene product expression or deliver transgene with higher activity (84); either approach would allow for dose sparing. At the same time the field should explore immunosuppressive regimens that selectively block B cell responses to AAV gene transfer. Preventing induction of AAV neutralizing antibodies following gene transfer by either inhibiting B cell activation or by removing such antibodies from the circulation would permit repeated administration of lower doses of AAV vectors to replace the currently used bolus approach (85).

## Author contributions

The author confirms being the sole contributor of this work and has approved it for publication.

## Conflict of interest

The author consults for several Gene Therapy companies and holds equity in Virion Therapeutics.

## Publisher’s note

All claims expressed in this article are solely those of the authors and do not necessarily represent those of their affiliated organizations, or those of the publisher, the editors and the reviewers. Any product that may be evaluated in this article, or claim that may be made by its manufacturer, is not guaranteed or endorsed by the publisher.

## References

[1] Maguire AM Simonelli F Pierce EA Pugh EN Mingozzi F Bennicelli J. Safety and efficacy of gene transfer for leber’s congenital amaurosis. N Engl J Med (2008) 358(21):2240–8. doi: 10.1056/NEJMoa0802315 PMC2829748 18441370

[2] Nathwani AC Tuddenham EGD Rangarajan S Rosales C McIntosh J Linch DC. Adenovirus-associated virus vector-mediated gene transfer in hemophilia b. N Engl J Med (2011) 365(25):2357–65. doi: 10.1056/NEJMoa1108046 PMC3265081 22149959

[3] Herzog RW Hagstrom JN Kung SH Tai SJ Wilson JM Fisher KJ. Stable gene transfer and expression of human blood coagulation factor IX after intramuscular injection of recombinant adeno-associated virus. Proc Natl Acad Sci U S A (1997) 94(11):5804–9. doi: 10.1073/pnas.94.11.5804 PMC20861 9159155

[4] Kumar SRP Xie J Hu S Ko J Huang Q Brown HC. Coagulation factor IX gene transfer to non-human primates using engineered AAV3 capsid and hepatic optimized expression cassette. Mol Ther - Methods Clin Dev (2021) 23:98–107. doi: 10.1016/j.omtm.2021.08.001 34631930 PMC8476648

[5] Niemeyer GP Herzog RW Mount J Arruda VR Tillson DM Hathcock J. Long-term correction of inhibitor-prone hemophilia b dogs treated with liver-directed AAV2-mediated factor IX gene therapy. Blood (2009) 113(4):797–806. doi: 10.1182/blood-2008-10-181479 18957684 PMC2630266

[6] Manno CS Pierce GF Arruda VR Glader B Ragni M Rasko JJ. Successful transduction of liver in hemophilia by AAV-factor IX and limitations imposed by the host immune response. Nat Med (2006) 12(3):342–7. doi: 10.1038/nm1358 16474400

[7] Mingozzi F Maus MV Hui DJ Sabatino DE Murphy SL Rasko JEJ. CD8+ T-cell responses to adeno-associated virus capsid in humans. Nat Med (2007) 13(4):419–22. doi: 10.1038/nm1549 17369837

[8] Fitzpatrick Z Leborgne C Barbon E Masat E Ronzitti G van Wittenberghe L. Influence of pre-existing anti-capsid neutralizing and binding antibodies on AAV vector transduction. Mol Ther Methods Clin Dev (2018) 9:119–29. doi: 10.1016/j.omtm.2018.02.003 PMC5948224 29766022

[9] Samelson-Jones BJ Finn JD Favaro P Wright JF Arruda VR. Timing of intensive immunosuppression impacts risk of transgene antibodies after AAV gene therapy in nonhuman primates. Mol Ther Methods Clin Dev (2020) 17:1129–38. doi: 10.1016/j.omtm.2020.05.001 PMC7256432 32490034

[10] Calcedo R Kuri-Cervantes L Peng H Qin Q Boyd S Schneider M. Immune responses in 101HEMB01, a phase 1/2 open-label, single ascending dose-finding trial of DTX101 (AAVrh10FIX) in patients with severe hemophilia b. Blood (2017) 130(Suppl):3333.

[11] High KA George LA Eyster ME Sullivan SK Ragni MV Croteau SE. A phase 1/2 trial of investigational spk-8011 in hemophilia a demonstrates durable expression and prevention of bleeds. Blood (2018) 132(Suppl):487. doi: 10.1182/blood-2018-99-115495

[12] Pipe S Stine K Rajasekhar A Everington T Poma A Crombez E. 101HEMB01 is a phase 1/2 open-label, single ascending dose-finding trial of DTX101 (AAVrh10FIX) in patients with moderate/severe hemophilia b that demonstrated meaningful but transient expression of human factor IX (hFIX). Blood (2017) 130(Suppl):3331.

[13] Srivastava A. *In vivo* tissue-tropism of adeno-associated viral vectors. Curr Opin Virol (2016) 21:75–80. doi: 10.1016/j.coviro.2016.08.003 27596608 PMC5138125

[14] Vercauteren K Hoffman BE Zolotukhin I Keeler GD Xiao JW Basner-Tschakarjan E. Superior *in vivo* transduction of human hepatocytes using engineered AAV3 capsid. Mol Ther (2016) 24(6):1042–9. doi: 10.1038/mt.2016.61 PMC4923326 27019999

[15] Li H Lasaro MO Jia B Lin SW Haut LH High KA. Capsid-specific T-cell responses to natural infections with adeno-associated viruses in humans differ from those of nonhuman primates. Mol Ther (2011) 19(11):2021–30. doi: 10.1038/mt.2011.81 PMC3222540 21587208

[16] Mingozzi F Meulenberg JJ Hui DJ Basner-Tschakarjan E Hasbrouck NC Edmonson SA. AAV-1-mediated gene transfer to skeletal muscle in humans results in dose-dependent activation of capsid-specific T cells. Blood (2009) 114(10):2077–86. doi: 10.1182/blood-2008-07-167510 PMC2744569 19506302

[17] Kumar H Kawai T Akira S. Pathogen recognition by the innate immune system. Int Rev Immunol (2011) 30(1):16–34. doi: 10.3109/08830185.2010.529976 21235323

[18] Kawai T Akira S. TLR signaling. Cell Death Differ (2006) 13(5):816–25. doi: 10.1038/sj.cdd.4401850 16410796

[19] Takeda K Akira S. Toll-like receptors. Curr Protoc Immunol (2015) 109:14.12.1–14.12.10. doi: 10.1002/0471142735.im1412s109 25845562

[20] Zolotukhin I Markusic DM Palaschak B Hoffman BE Srikanthan MA Herzog RW. Potential for cellular stress response to hepatic factor VIII expression from AAV vector. Mol Ther Methods Clin Dev (2016) 3:16063. doi: 10.1038/mtm.2016.63 27738644 PMC5040172

[21] Dunkelberger JR Song WC. Complement and its role in innate and adaptive immune responses. Cell Res (2010) 20(1):34–50. doi: 10.1038/cr.2009.139 20010915

[22] Merle NS Church SE Fremeaux-Bacchi V Roumenina LT. Complement system part I - molecular mechanisms of activation and regulation. Front Immunol (2015) 6:262. doi: 10.3389/fimmu.2015.00262 26082779 PMC4451739

[23] Kumar NA Kunnakkadan U Thomas S Johnson JB. In the crosshairs: RNA viruses or complement? Front Immunol (2020) 11:573583. doi: 10.3389/fimmu.2020.573583 33133089 PMC7550403

[24] Gorbet MB Sefton MV. Biomaterial-associated thrombosis: Roles of coagulation factors, complement, platelets and leukocytes. Biomaterials (2004) 25(26):5681–703. doi: 10.1016/j.biomaterials.2004.01.023 15147815

[25] Merle NS Noe R Halbwachs-Mecarelli L Fremeaux-Bacchi V Roumenina LT. Complement system part II: Role in immunity. Front Immunol (2015) 6:257. doi: 10.3389/fimmu.2015.00257 26074922 PMC4443744

[26] Kerr H Richards A. Complement-mediated injury and protection of endothelium: Lessons from atypical haemolytic uraemic syndrome. Immunobiology (2012) 217(2):195–203. doi: 10.1016/j.imbio.2011.07.028 21855165 PMC4083254

[27] Subramaniam S Jurk K Hobohm L Jäckel S Saffarzadeh M Schwierczek K. Distinct contributions of complement factors to platelet activation and fibrin formation in venous thrombus development. Blood (2017) 129(16):2291–302. doi: 10.1182/blood-2016-11-749879 PMC5399485 28223279

[28] Ahmad A Mandwie M Dreismann AK Smyth CM Doyle H Malik TH. Adeno-associated virus vector gene delivery elevates factor I levels and downregulates the complement alternative pathway *In vivo* . Hum Gene Ther (2021) 32(21–22):1370–81. doi: 10.1089/hum.2021.022 34238030

[29] Akkaya M Kwak K Pierce SK. B cell memory: building two walls of protection against pathogens. Nat Rev Immunol (2020) 20(4):229–38. doi: 10.1038/s41577-019-0244-2 PMC7223087 31836872

[30] Allman D Pillai S. Peripheral b cell subsets. Curr Opin Immunol (2008) 20(2):149–57. doi: 10.1016/j.coi.2008.03.014 PMC2532490 18434123

[31] Cyster JG Allen CDC. B cell responses: cell interaction dynamics and decisions. Cell (2019) 177(3):524–40. doi: 10.1016/j.cell.2019.03.016 PMC6538279 31002794

[32] Ruterbusch M Pruner KB Shehata L Pepper M. *In vivo* CD4+ T cell differentiation and function: revisiting the Th1/Th2 paradigm. Annu Rev Immunol (2020) 38:705–25. doi: 10.1146/annurev-immunol-103019-085803 32340571

[33] Zhu J Yamane H Paul WE. Differentiation of effector CD4 T cell populations (*). Annu Rev Immunol (2010) 28:445–89. doi: 10.1146/annurev-immunol-030409-101212 PMC3502616 20192806

[34] Cruz FM Colbert JD Merino E Kriegsman BA Rock KL. The biology and underlying mechanisms of cross-presentation of exogenous antigens on MHC-I molecules. Annu Rev Immunol (2017) 35:149–76. doi: 10.1146/annurev-immunol-041015-055254 PMC5508990 28125356

[35] Manz BN Jackson BL Petit RS Dustin ML Groves J. T-Cell triggering thresholds are modulated by the number of antigen within individual T-cell receptor clusters. Proc Natl Acad Sci USA (2011) 108(22):9089–94. doi: 10.1073/pnas.1018771108 PMC3107331 21576490

[36] Palmer CS Ostrowski M Balderson B Christian N Crowe SM. Glucose metabolism regulates T cell activation, differentiation, and functions. Front Immunol (2015) 6:1. doi: 10.3389/fimmu.2015.00001 25657648 PMC4302982

[37] Jameson SC Masopust D. Understanding subset diversity in T cell memory. Immunity (2018) 48(2):214–26. doi: 10.1016/j.immuni.2018.02.010 PMC5863745 29466754

[38] Beltra JC Manne S Abdel-Hakeem MS Kurachi M Giles JR Chen Z. Developmental relationships of four exhausted CD8+ T cell subsets reveals underlying transcriptional and epigenetic landscape control mechanisms. Immunity (2020) 52(5):825–41. doi: 10.1016/j.immuni.2020.04.014 PMC8360766 32396847

[39] Wherry EJ Blattman JN Murali-Krishna K van der Most R Ahmed R. Viral persistence alters CD8 T-cell immunodominance and tissue distribution and results in distinct stages of functional impairment. J Virol (2003) 77(8):4911–27. doi: 10.1128/JVI.77.8.4911-4927.2003 PMC152117 12663797

[40] Kim J Kim AR Shin EC. Cytomegalovirus infection and memory T cell inflation. Immune Netw (2015) 15(4):186–90. doi: 10.4110/in.2015.15.4.186 PMC4553256 26330804

[41] Flynn K Müllbacher A. Memory alloreactive cytotoxic T cells do not require costimulation for activation *in vitro* . Immunol Cell Biol (1996) 74(5):413–20. doi: 10.1038/icb.1996.71 8912004

[42] Mehlhop-Williams ER Bevan MJ. Memory CD8+ T cells exhibit increased antigen threshold requirements for recall proliferation. J Exp Med (2014) 211(2):345–56. doi: 10.1084/jem.20131271 PMC3920562 24493801

[43] Whitehead M Osborne A Yu-Wai-Man P Martin K. Humoral immune responses to AAV gene therapy in the ocular compartment. Biol Rev Camb Philos Soc (2021) 96(4):1616–44. doi: 10.1111/brv.12718 33837614

[44] Zheng M Tian Z. Liver-mediated adaptive immune tolerance. Front Immunol (2019) 10:2525. doi: 10.3389/fimmu.2019.02525 31787967 PMC6856635

[45] Oh SJ Lee JK Shin OS. Immunogenicity aging and the immune system: the impact of immunosenescence on viral infection, immunity. Immune Netw (2019) 19(6):e37. doi: 10.4110/in.2019.19.e37 31921467 PMC6943173

[46] Araki K Turner AP Shaffer VO Gangappa S Keller SA Bachmann MF. mTOR regulates memory CD8 T-cell differentiation. Nature (2009) 460(7251):108–12. doi: 10.1038/nature08155 PMC2710807 19543266

[47] Ferrer IR Araki K Ford ML. Paradoxical aspects of rapamycin immunobiology in transplantation. Am J Transplant (2011) 11(4):654–9. doi: 10.1111/j.1600-6143.2011.03473.x PMC3685502 21446969

[48] Xiang Z Kurupati RK Li Y Kuranda K Zhou X Mingozzi F. The effect of CpG sequences on capsid-specific CD8+ T cell responses to AAV vector gene transfer. Mol Ther (2020) 28(3):771–83. doi: 10.1016/j.ymthe.2019.11.014 PMC7054717 31839483

[49] Zhu J Huang X Yang Y. The TLR9-MyD88 pathway is critical for adaptive immune responses to adeno-associated virus gene therapy vectors in mice. J Clin Invest (2009) 119(8):2388–98. doi: 10.1172/JCI37607 PMC2719948 19587448

[50] Hösel M Broxtermann M Janicki H Esser K Arzberger S Hartmann P. Toll-like receptor 2-mediated innate immune response in human nonparenchymal liver cells toward adeno-associated viral vectors. Hepatology (2012) 55(1):287–97. doi: 10.1002/hep.24625 21898480

[51] Shao W Earley LF Chai Z Chen X Sun J He T. Double-stranded RNA innate immune response activation from long-term adeno-associated virus vector transduction. JCI Insight (2018) 3(12):e120474. doi: 10.1172/jci.insight.120474 PMC6124417 29925692

[52] Bertolini TB Shirley JL Zolotukhin I Li X Kaisho T Xiao W. Effect of CpG depletion of vector genome on CD8+ T cell responses in AAV gene therapy. Front Immunol (2021) 12:672449. doi: 10.3389/fimmu.2021.672449 34135899 PMC8200677

[53] Faust SM Bell P Cutler BJ Ashley SN Zhu Y Rabinowitz JE. CpG-depleted adeno-associated virus vectors evade immune detection. J Clin Invest (2013) 123(7):2994–3001. doi: 10.1172/JCI68205 23778142 PMC3696560

[54] Pan X Yue Y Boftsi M Wasala LP Tran NT Zhang K. Rational engineering of a functional CpG-free ITR for AAV gene therapy. Gene Ther (2022) 29(6):333–45. doi: 10.1038/s41434-021-00296-0 PMC8983793 34611321

[55] Milligan ID Gibani MM Sewell R Clutterbuck EA Campbell D Plested E. Safety and immunogenicity of novel adenovirus type 26- and modified vaccinia Ankara-vectored Ebola vaccines: A randomized clinical trial. JAMA (2016) 315(15):1610–23. doi: 10.1001/jama.2016.4218 27092831

[56] Teichler Zallen D. US Gene therapy in crisis. Trends Genet (2000) 16(6):272–5. doi: 10.1016/S0168-9525(00)02025-4 10827455

[57] Chand DH Zaidman C Arya K Millner R Farrar MA Mackie FE. Thrombotic microangiopathy following onasemnogene abeparvovec for spinal muscular atrophy: A case series. J Pediatr (2021) 231:265–8. doi: 10.1016/j.jpeds.2020.11.054 33259859

[58] Mendell JR Al-Zaidy SA Rodino-Klapac LR Goodspeed K Gray SJ Kay CN. Current clinical applications of *in vivo* gene therapy with AAVs. Mol Ther (2021) 29(2):464–88. doi: 10.1016/j.ymthe.2020.12.007 PMC7854298 33309881

[59] Mullard A. Gene therapy community grapples with toxicity issues, as pipeline matures. Nat Rev Drug Discov (2021) 20(11):804–5. doi: 10.1038/d41573-021-00164-x 34599291

[60] Kok RH Wolfhagen MJ Klosters G. A syndrome resembling thrombotic thrombocytopenic purpura associated with human parvovirus B19 infection. Clin Infect Dis (2001) 32(2):311–2. doi: 10.1086/318481 11170925

[61] Palma LMP Sridharan M Sethi S. Complement in secondary thrombotic microangiopathy. Kidney Int Rep (2021) 6(1):11–23. doi: 10.1016/j.ekir.2020.10.009 33102952 PMC7575444

[62] Niederreiter J Eck C Ries T Hartmann A Märkl B Büttner-Herold M. Complement activation *via* the lectin and alternative pathway in patients with severe COVID-19. Front Immunol (2022) 13:835156. doi: 10.3389/fimmu.2022.835156 35237273 PMC8884149

[63] Asokan A Johnson JS Li C Samulski RJ. Bioluminescent virion shells: New tools for quantitation of AAV vector dynamics in cells and live animals. Gene Ther (2008) 15(24):1618–22. doi: 10.1038/gt.2008.127 PMC2684674 18668144

[64] Selot R Arumugam S Mary B Cheemadan S Jayandharan GR. Optimized AAV rh.10 vectors that partially evade neutralizing antibodies during hepatic gene transfer. Front Pharmacol (2017) 8:441. doi: 10.3389/fphar.2017.00441 28769791 PMC5511854

[65] Tse LV Klinc KA Madigan VJ Castellanos Rivera RM Wells LF Havlik LP. Structure-guided evolution of antigenically distinct adeno-associated virus variants for immune evasion. Proc Natl Acad Sci USA (2017) 114(24):E4812–21. doi: 10.1073/pnas.1704766114 PMC5474820 28559317

[66] Mingozzi F Anguela XM Pavani G Chen Y Davidson RJ Hui DJ. Overcoming preexisting humoral immunity to AAV using capsid decoys. Sci Transl Med (2013) 5(194):194ra92. doi: 10.1126/scitranslmed.3005795 PMC4095828 23863832

[67] Orlowski A Katz MG Gubara SM Fargnoli AS Fish KM Weber T. Successful transduction with AAV vectors after selective depletion of anti-AAV antibodies by immunoadsorption. Mol Ther - Methods Clin Dev (2020) 16:192–203. doi: 10.1016/j.omtm.2020.01.004 32055647 PMC7011017

[68] Leborgne C Barbon E Alexander JM Hanby H Delignat S Cohen DM. IgG-cleaving endopeptidase enables *in vivo* gene therapy in the presence of anti-AAV neutralizing antibodies. Nat Med (2020) 26(7):1096–101. doi: 10.1038/s41591-020-0911-7 32483358

[69] Ros-Gañán I Hommel M Trigueros-Motos L Tamarit B Rodríguez-García E Salas D. Optimising the IgG-degrading enzyme treatment regimen for enhanced adeno-associated virus transduction in the presence of neutralising antibodies. Clin Transl Immunol (2022) 11(2):e1375. 10.1002/cti2.1375 PMC8867416 35228870

[70] Cao O Dobrzynski E Wang L Nayak S Mingle B Terhorst C. Induction and role of regulatory CD4+CD25+ T cells in tolerance to the transgene product following hepatic *in vivo* gene transfer. Blood (2007) 110(4):1132–40. doi: 10.1182/blood-2007-02-073304 PMC1939896 17438084

[71] Nayak S Sarkar D Perrin GQ Moghimi B Hoffman BE Zhou S. Prevention and reversal of antibody responses against factor IX in gene therapy for hemophilia b. Front Microbiol (2011) 2:244. doi: 10.3389/fmicb.2011.00244 22279442 PMC3260742

[72] Xiang Z Kuranda K Quinn W Chekaoui A Ambrose R Hasanpourghai M. The effect of rapamycin and ibrutinib on antibody responses to adeno-associated virus vector-mediated gene transfer. Hum Gene Ther (2022) 33(11–12):614–24. doi: 10.1089/hum.2021.258 35229644

[73] George LA Monahan PE Eyster ME Sullivan SK Ragni MV Croteau SE. Multiyear factor VIII expression after AAV gene transfer for hemophilia a. N Engl J Med (2021) 385(21):1961–73. doi: 10.1056/NEJMoa2104205 PMC8672712 34788507

[74] FDA. BRIEFING DOCUMENT: Food and drug administration (FDA) cellular, tissue, and gene therapies advisory committee (CTGTAC) meeting #70; toxicity risks of adeno-associated virus (AAV) vectors for gene therapy (GT) (2021). Available at: https://www.fda.gov/media/151599/download.

[75] Day JW Mendell JR Mercuri E Finkel RS Strauss KA Kleyn A. Clinical trial and postmarketing sSafety of onasemnogene abeparvovec therapy. Drug Saf (2021) 44(10):1109–19. doi: 10.1007/s40264-021-01107-6 PMC8473343 34383289

[76] Tokunaga A Sugiyama D Maeda Y Warner AB Panageas KS Ito S. Selective inhibition of low-affinity memory CD8+ T cells by corticosteroids. J Exp Med (2019) 216(12):2701–13. doi: 10.1084/jem.20190738 PMC6888983 31537643

[77] Nonomura A Mizukami Y Matsubara F Nakanuma Y Kobayashi K. The cellular infiltrate in the liver of patients with fulminant hepatitis: Analysis of paraffin-embedded tissue sections. Intern Med (1992) 31(2):154–9. doi: 10.2169/internalmedicine.31.154 1600259

[78] Hinderer C Katz N Buza EL Dyer C Goode T Bell P. Severe toxicity in nonhuman primates and piglets following high-dose intravenous administration of an adeno-associated virus vector expressing human SMN. Hum Gene Ther (2018) 29(3):285–98. doi: 10.1089/hum.2018.015 PMC5865262 29378426

[79] Hordeaux J Buza EL Jeffrey B Song C Jahan T Yuan Y. MicroRNA-mediated inhibition of transgene expression reduces dorsal root ganglion toxicity by AAV vectors in primates. Sci Transl Med (2020) 12(569):eaba9188. doi: 10.1126/scitranslmed.aba9188 33177182

[80] Mueller C Berry JD McKenna-Yasek DM Gernoux G Owegi MA Pothier LM. SOD1 suppression with adeno-associated virus and microRNA in familial ALS. N Engl J Med (2020) 383(2):151–8. doi: 10.1056/NEJMoa2005056 PMC11836664 32640133

[81] Sherafat R AC Planning Working Group Office of Tissues and Advanced Therapies (OTAT) CBER FDA. Food and drug administration (FDA) cellular, tissue, and gene therapies advisory committee (CTGTAC) meeting #70; toxicity risks of adeno-associated virus (AAV) vectors for gene therapy (GT) . Available at: https://www.fda.gov/media/151969/download.

[82] Whiteley L Hordeaux J. rAAV gene therapy and peripheral nervous system ganglia toxicity . Available at: https://asgct.org/global/documents/advocacy/2021-fda-liaison-meeting/redacted-final-drg-asgct-fda-nov-2021.aspx.

[83] Tripodi L Villa C Molinaro D Torrente Y Farini A. The immune system in duchenne muscular dystrophy pathogenesis. Biomedicines (2021) 9(10):1447. doi: 10.3390/biomedicines9101447 34680564 PMC8533196

[84] VandenDriessche T Chuah MK. Hyperactive factor IX padua: a game-changer for hemophilia gene therapy. Mol Ther (2018) 26(1):14–6. doi: 10.1016/j.ymthe.2017.12.007 PMC5763158 29274719

[85] Kishimoto TK Samulski RJ. Addressing high dose AAV toxicity - “one and done” or “slower and lower”? Expert Opin Biol Ther (2022), 3:1–5. doi: 10.1080/14712598.2022.2060737 35373689

